# Reconstructing Somalia’s population: A district level analysis

**DOI:** 10.1371/journal.pgph.0005215

**Published:** 2025-09-25

**Authors:** Yamna Ouchtar, Dahir Abdi Ali, Yahye Abukar Ahmed, Francesco Checchi

**Affiliations:** 1 Department of Infectious Disease Epidemiology, Faculty of Epidemiology and Population Health, London School of Hygiene and Tropical Medicine, London, United Kingdom; 2 Faculty of Economics, SIMAD University, Mogadishu, Somalia; 3 Faculty of Computing, SIMAD University, Mogadishu, Somalia; Nanyang Technological University, SINGAPORE

## Abstract

Somalia has experienced more than 30 years of armed conflict exacerbated by drought and food insecurity, which has led to major migratory flows. Despite these large-scale movements, no census has been carried out since 1975. To support effective planning and service delivery, we reconstructed Somalia’s population at district level by taking into account alternative sources of population data, natural growth and known internal and refugee displacement flows.A previous study, published in PLOS Global Public Health, attempted to reconstruct the population of Somalia by district (administrative level 2) on a monthly basis from 2013 to 2022. This initial method was based on the average of available estimates, on the assumption of a fixed rate of natural increase and on the allocation of displaced persons to the various districts. However, it assumed that internally displaced persons (IDPs) would remain in their destination districts indefinitely, leading to unrealistic population declines and inflated IDP numbers. The paper presents an improved reconstruction method using mechanistic and statistical models to overcome these limitations.The updated method incorporates dynamic modelling techniques, reflecting more realistic migration and displacement patterns. The new model indicates that previous estimates significantly underestimated populations in some districts. The revised estimates provide a more balanced distribution, reducing instances of implausibly high or negative population figures. For example, districts thought to be almost depopulated are revealed to have more viable population levels.Key advances include the use of probabilistic rates of return for displaced people and the integration of new data sources, allowing for a more accurate representation of population movements. These results provide a more reliable basis for planning and service delivery, accurately reflecting the impacts of conflict and climate-induced displacement between 2013 and 2024. The improved model presents a nuanced reconstruction of Somalia’s population dynamics, essential for informed decision-making.

## Introduction

Somalia has experienced more than three decades of armed conflict which, combined with recurrent droughts and severe food insecurity, have led to major population displacements. Since the outbreak of civil war in the early 1990s, millions of Somalis have been forced to flee their homes, either seeking refuge in neighbouring countries or becoming internally displaced persons. Over the past fifteen years, an unprecedented series of four serious droughts has led to further waves of displacement [1,2].

Despite the profound impact of these population movements, Somalia has not conducted a national demographic census since 1975. This prolonged absence of comprehensive demographic data poses considerable challenges to governance, planning and the provision of essential services. Accurate demographic data are also essential to design, adequately resource and evaluate humanitarian responses, particularly in areas where there is a great deal of displacement [3].

Demographic projections at country level are relatively simple if one assumes a certain rate of natural increase and adjusts resulting estimates for known refugee or voluntary migration as well as returns [4]. However, at sub-national level, demographic uncertainties are much greater due to complex patterns of migration and displacement.

We previously attempted to reconstruct the evolution of Somalia’s population by district (administrative level 2) and month so as to convert estimates of death rate attributable to drought crises into (excess) death tolls [5,6]. We did so by averaging four alternative base sources of district-level population based on their relative robustness, assuming a fixed natural growth rate and subtracting or adding newly internally displaced persons (IDPs) from and to districts on the basis of available information (see Methods). However, a major limitation of this approach is the assumption that IDPs remain indefinitely in their arrival districts, which over time leads to unrealistic population declines in some districts (including negative populations) and IDP numbers exceeding total population figures.

This paper presents an improved method for reconstructing the population of Somalia, which addresses the limitations of the previous approach through the use of mechanistic and statistical modelling that explicitly accounts for (partial) returns to districts of origin.

## Methods

### Study population and period

For this analysis, Somalia includes Somaliland and Puntland, reflecting the country’s borders prior to 1991. We refer to the 74 districts and their boundaries as commonly used by the United Nations. Banadir region, which contains the capital, Mogadishu, and which is itself divided into several urban districts, is treated as a single large district, mainly because none of the sources of demographic information available to us present data below the regional level for Banadir. We estimated population (all ages and under 5 years old) by month over the period July 2013 to December 2024.

### Data sources

#### Cross-sectional population estimates.

Disaggregated, point-in-time population estimates by district or lower level were available from four sources: (i) **AfriPop/WorldPop** (*January 2015* - https://www.worldpop.org/): A global project providing high-resolution gridded population estimates (typically 100m^2^ pixel size). These estimates are derived from a validated statistical predictive model that integrates census data with a range of geospatial covariates (e.g., land cover, night-time lights, road networks) primarily obtained through remote sensing. Somalia served as key case study during the development and validation of this methodology [7,8]; (ii) **Expanded Programme on Immunisation** (EPI) planning data (*January 2019*), based on national census projections but adjusted using immunisation campaign coverage data to improve accuracy in areas with incomplete or outdated census figures. These adjustments account for observed discrepancies between expected and actual vaccinated populations and are then extrapolated to district-level general population estimates using standard age–sex distribution assumptions; (iii) **Global Polio Eradication Initiative Data** (*December 2018*) consisting of district-level target population denominators derived for polio catch-up campaigns. These estimates are periodically updated through active household-level enumeration exercises conducted by implementing partners, providing a more granular understanding of actual population distribution; (iv) the **United Nations Population Estimation Sample Survey** (UNPESS) (*March 2014*), a survey designed to address gaps in census for Somalia. It collected detailed household demographic data from a stratified sample across urban, rural, IDP, and nomadic communities. Results were used to adjust crude census projections by incorporating observed household size, mobility, and settlement type characteristics [9].

**Fig 1:**
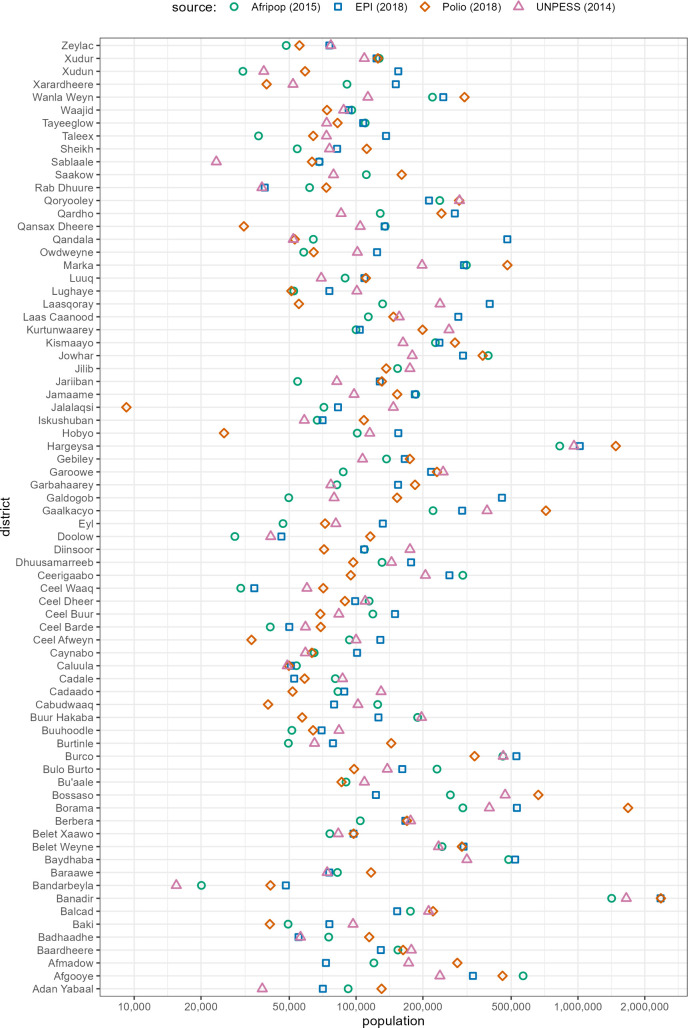
Estimates of district population, by available base source. Note that population is presented on a logarithmic (base 2) scale for easier readability.

As shown in Fig 1, considerable district-level discrepancies were evident when comparing the four sources. Moreover, the 2018 polio and 2019 EPI sources exclude Baydhaba, Bu’aale, Jilib, and Saakow districts. After reviewing all documentation associated with each source, we attributed a quality score from 0 to 1 to each using published criteria that consider the theoretical robustness of the method, the estimate’s precision, the extent of potential bias, the expertise/ credibility of the source’s authors and the estimate’s plausibility [10].

#### Proportion of the population that is aged under 5 years.

Standardised Monitoring of Relief and Transition (SMART) anthropometric and mortality surveys, described in detail elsewhere [5], are routinely conducted across Somalia and in other humanitarian responses to monitor nutritional status and retrospective mortality, typically at administrative level 3 or comparable scale, with sample sizes of about 500–1000 households selected through multi-stage probability-proportional-to-size cluster sampling. Each survey records the number of people of all ages and under five years of age in households surveyed. We also had access to raw data from 496 surveys conducted in Somalia between 2013 and 2024, covering 53 districts with a combined sample of 163,166 households. These surveys provide key demographic indicators, including under-five mortality rates. In addition, the 2020 Somali Health and Demographic Survey (SHDS) estimates that approximately 20.5% of the population is under the age of five [11].

#### Natural growth rate.

A constant annual growth rate of 1.96%, derived from the difference between the crude birth rate estimated by the SHDS 2020 and the crude death rate estimated for non-crisis periods between 2013 and 2022, was assumed to back- and forward-calculate population based on the four alternative sources mentioned above.

#### Displacement data.

Population displacement in Somalia involves two main categories: (i) refugees, who cross international borders, and (ii) IDPs, who remain within Somalia. Displacement can be described in terms of flows (movements over time, such as new displacements or returns) or prevalence (the stock of displaced people present in a location at a given time). In Somalia, district-to-district movements, including potential returns to districts of origin, are tracked by the United Nations High Commissioner for Refugees (UNHCR)-led Protection and Returns Monitoring Network (PRMN) [12] (https://raw.githubusercontent.com/unhcr/dataviz-somalia-prmn/), with monthly data publicly available from January 2016. PRMN tracks flow of IDPS between district, including returns. Archived data prior to 2016 were not made available to us. Accordingly, we assumed no movement of IDPs from April 2014 to December 2015, a period considered to be relatively stable in security terms and during which drought conditions were not observed.

Humanitarian situation reports from ReliefWeb (www.reliefweb.int) and UNHCR (www.unhcr.org) were reviewed to estimate refugee flows by district, cross-border movements and returns, up to December 2018, with the exception of Laas Canood, where 80,000 people are assumed to have crossed into Ethiopia in February 2023. Refugee flows after 2018 are considered numerically negligible based on countrywide data available from UNHCR.

Data on prevalent internal displacement came from the March 2014 UNPESS survey, which estimated the percentage of displaced people in each district. The Displacement Tracking Matrix (DTM) project of the International Organization for Migration (IOM) also tracks displacement. The DTM has carried out cross-sectional assessments of IDP and returnee settlements through field visits or remote contacts with local informants. All DTM assessments including both displaced persons and returnees, available as of 05 April 2025 on the www.dtm.iom.int/datasets website, were used. These assessments detail return periods, district-level data, and the number and rate (by month) of returnees.

#### Predictors of displacement.

Predictors that could predict a district’s suitability for IDP return were added to the DTM returnee dataset. These predictors included the average rate of insecurity-related events (events per month), the rate of insecurity-related deaths (deaths per month) [13], terms of trade [14] (wages per cereal and goats per cereal) and rainfall [15] (standard rainfall index or SPI, indicating the deviation from the historical average) by district and assessment period. Each predictor was centred and scaled for continuous use and categorised as required [5].

### Population and displacement model

**Fig 2:**
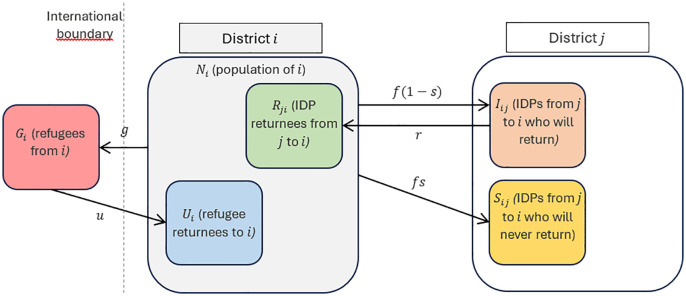
Schematic of compartmental model to reconstruct population.

A mechanistic model of population growth and movement was defined, illustrated simplistically in Fig 2 for a pair of districts. For readability, only displacement from district i is displayed, but it is implicit that district i may also receive IDPs from district j; i and j may also represent the same district if IDPs do not cross a district boundary.

Available literature [16] suggests that a proportions of IDPs settle in their district of arrival and never return, while the remainder eventually go back. The total rate of IDP movement can thus be expressed as f=fs+f(1−s). Evidence on when the proportion 1−s returns to their community of origin, or how time since displacement affects the rate of return r, is lacking. To simplify computation and in the absence of a more plausible pattern, we assumed that the time to return follows a negative exponential decay distribution with r as the time-independent rate of return; accordingly, $\frac{\text{1}}{r}$ is the mean duration of displacement conditional on eventually returning.

The following equations describe the evolution of population within any district i∈{1,2…K} over any time step t∈{1,2…T} (month in our case):


$$N_{i,t+\text{1}}=(\text{1}+b-\mu )N_{i,t}-\sum_{j=\text{1}}^{j=K}f_{ij,t}+\sum_{j=\text{1}}^{j=K}f_{ji,t}-\sum_{j=\text{1}}^{j=K}{I_{ji,t}r}_{ij}+{\sum_{j=\text{1}}^{j=K}{I_{ij,t}r}_{ji}-G}_{i,t}+U_{i,t}$$

where the right-hand terms are, respectively, natural growth (birth rate b minus death rate μ); new IDPs from district i moving to any j of the K possible districts (including i itself); new IDPs arriving to i from any district, including i itself; IDPs in i who return to their districts of origin; IDPs returning to i from any districts; new refugees; and returning refugees. Note that, in accordance with what data are available data for Somalia, f, G and U are absolute numbers per time unit, while b, μ and r are treated as per-capita rates.

The displacement compartments evolve as follows:


$$I_{i,t+\text{1}}=(\text{1}+b-\mu )I_{i,t}+\sum_{j=\text{1}}^{j=K}f_{ji,t}(\text{1}-s_{ji})-\sum_{j=\text{1}}^{j=K}{I_{ji,t}r}_{ij}$$

$$S_{i,t+\text{1}}=(\text{1}+b-\mu )S_{i,t}+\sum_{j=\text{1}}^{j=K}f_{ji,t}s_{ji}$$

$$R_{i,t+\text{1}}=(\text{1}+b-\mu )R_{i,t}+\sum_{j=\text{1}}^{j=K}{I_{ij,t}r}_{ji}$$

The proportion of IDPs in any district is thus ${p_{i,t}=(I}_{i,t}+S_{i,t})/N_{i,t}$.

The following simplifying assumptions are made:

- The birth and death rates are equal and constant across time, space and the different population compartments. This assumption could be relaxed but is unlikely to substantively affect overall population estimates.
- There is no onward displacement, i.e. IDPs only move from one district to another or back. Relaxing this assumption would be straightforward if data on onward movements were available.

### Estimation of IDP return parameters

We quantified the goodness-of-fit of candidate values of s (the proportion of IDPs who never return) and 1/r (the mean time to return in months, among IDPs who do) for three large strata consisting of (i) Puntland and Somaliland, relatively secure areas of Somalia where displacement appears to be a long-term phenomenon largely driven by food insecurity; (ii) ‘South-Central 1’ (Bakool, Banadir, Bay, Lower Shabelle and Middle Shabelle regions), where urban Baydhaba, Mogadishu and the neighbouring Afgooye corridor are the major destinations of displacement; and (iii) ‘South-Central 2’ (Galgaduud, Gedo, Hiraan, Lower Juba, Middle Juba). We defined these strata so as to provide at least some geographic specificity, in the absence of district-level predictive models (see below).

A grid of candidate values of s (from 0 to 1) and 1/r (from 0 to 120mths, i.e., 0–10yrs) was explored, with a Latin hypercube sample of 1000 sets of both values chosen randomly within this grid for each geographic stratum. For each candidate parameter value set and for each of the four population estimate sources, population was forward- and back-computed from the time point that the estimate referred to, based on the equations above. The model-predicted proportion of returnees among all returnees plus IDPs (${\hat{\varphi }}_{i,t}={\hat{R}}_{i,t}/({\hat{R}}_{i,t}+{\hat{I}}_{i,t}+{\hat{S}}_{i,t}$)) was then compared to the observed proportion during cross-sectional assessments by the DTM. This estimator was chosen on the assumption that a proportion would be less sensitive to measurement bias than absolute figures, particularly when the DTM was unable to fully cover hard-to-reach districts.

**Fig 3:**
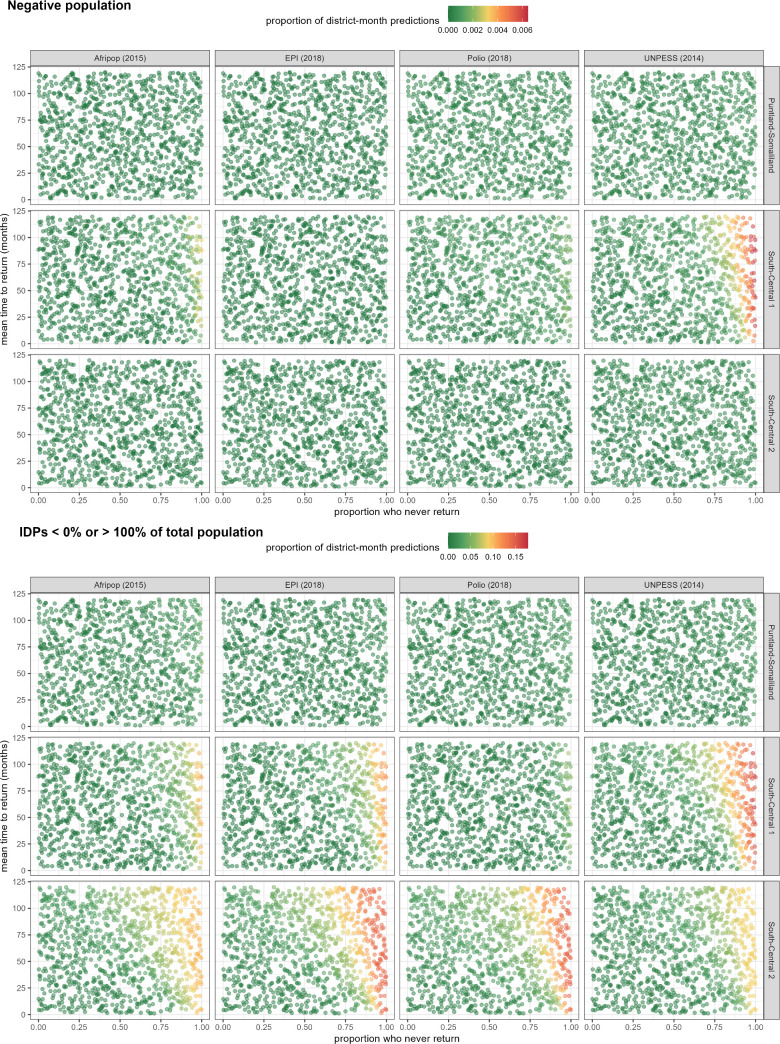
Proportion of district-month predictions with implausible values (top panel: negative population; bottom panel: proportion of IDPs out of total population <0% or > 100%), by population source and stratum. Each dot represents a candidate set of s and 1/r parameter values.

As a metric of goodness-of-fit, the negative log-likelihood was initially quantified as a weighted sum of the log binomial densities of the predicted $\hat{\phi }$, given the observed ϕ, over each of the V (n = 57) instances of district-month prevalent returnee and IDP data isolated from the DTM dataset, i.e., the fitting data:


$$-\ell (s,r)=\sum_{v=\text{1}}^{v=V}{-w}_{v}\text{log}\text{Bin}(\text{n}={\hat{R}}_{v},\text{trials}={\hat{R}}_{v}+{\hat{I}}_{v}+{\hat{S}}_{v},\;\text{prob}=\varphi_{v})$$

where each weight ${w_{v}=\left(\frac{\text{1}}{V}\sum_{v=\text{1}}^{v=V}\left(R_{v}+I_{v}+S_{v}\right)\right)}^{-\text{1}}\;$balances differences in the denominator (number of returnees and IDPs) among the DTM observations. The resulting likelihood profiles were relatively uninformative towards the minima of the parameter grid, and, towards its maxima, yielded increasingly frequent predictions with either negative population values or a number of IDPs > the total population (Fig 3). We therefore added logarithmic barrier penalties λ to the likelihoods for extreme candidate parameter values, yielding the following penalised negative log-likelihood [17]:

$-\ell_{\text{pen}}(s,r)=-\ell (s,r)+\lambda_{s}+\lambda_{r}$, where

$\lambda_{s}=\left\{ \;\;\;\;\begin{array}{c} -\text{log}(\frac{s-s_{\text{min}}}{b_{s,\text{lower}}-s_{\text{min}}}\backslash \;\text{if}\;s<b_{s,\text{lower}}\\ -\text{log}\left(\text{1}-\frac{s_{\text{max}}-s}{{s_{\text{max}}-b}_{s,\text{higher}}}\right)\;\text{if}\;s>b_{s,\text{higher}} \end{array}\right.\;$and


$$\lambda_{r}=\left\{ \;\;\;\;\begin{array}{c} -\text{log}\left(\frac{r-r_{\text{min}}}{b_{r,\text{lower}}-r_{\text{min}}}\right)\;\text{if}\;r<b_{r,\text{lower}}\\ -\text{log}\left(\text{1}-\frac{r_{\text{max}}-r}{{r_{\text{max}}-b}_{r,\text{higher}}}\right)\;\text{if}\;r>b_{r,\text{higher}} \end{array}\right.\;$$

The barrier values were set to $\left\{b_{s,\text{lower}}=\text{0}.\text{1}\text{0},b_{s,\text{higher}}=\text{0}.\text{7}\text{5}\right\}\;$ and $\left\{b_{r,\text{lower}}={\text{6}\text{mths}}^{-\text{1}},b_{r,\text{higher}}={\text{7}\text{5}\text{mths}}^{-\text{1}}\right\}$.

The two-dimensional likelihood surfaces obtained for each population source and stratum by fitting a generalised additive model to $-\ell_{\text{pen}}(s,r)$ were smoothed with a tensor product of s and r as the single predictor. This approach provided a continuous surface of plausible values rather than a single point estimate, allowing for the identification of the joint minima of the surface as the best-fitting parameters, while retaining the surrounding likelihood structure. By reporting this distribution of values (s, r), we were able to propagate the uncertainty of the parameters directly into the reconstructed population estimates.

### Estimation of the proportion of children under 5-years

Given the importance of the 5-year age threshold in public health applications, the proportion of the total population aged under 5 ($\pi_{\text{u}\text{5},i,t}$) was estimated by fitting a generalised additive quasi-binomial model to observed age group data from SMART household surveys. Predictors included survey period and a random effect term for district coverage. In addition, SHDS point estimation was used as an alternative.

### Population reconstruction

Population estimates, along with the number and proportion of IDPs and other relevant indicators, were generated through 1000 stochastic model runs. Each run involved random selection of a base population estimate among the four alternative sources, with selection probability proportional to each source’s quality score; a set of s and r values (with probability: the smoothed ${-\ell_{\text{pen}}(s,r)}^{-\text{1}}$); and a proportion of children aged under 5 years (from a normal distribution given by the point estimate and standard error estimated above for each district-year, back-transformed from logit to linear form). Mean, median, and 95th percentile values were computed across all runs.

### Ethics

Ethics approval was provided by the Ethics Committee of the LSHTM (ref. 15334) and the Ethics Committee of the Ministry of Health and Human Services of Somalia (ref. MOH&HS/DGO/1485/November/2022). All analyses in this paper were done in R [18] using packages bkmr for BKMR regression [19], glmmTMB for mixed linear models [20], lhs for Latin hypercube sampling and gamlss [21] or mgcv [22] for additive modelling. All data and analysis scripts, as well as reconstructed population denominators, are available from the authors and will be published on GitHub.

## Results

### Estimates of the IDP return parameters

**Fig 4:**
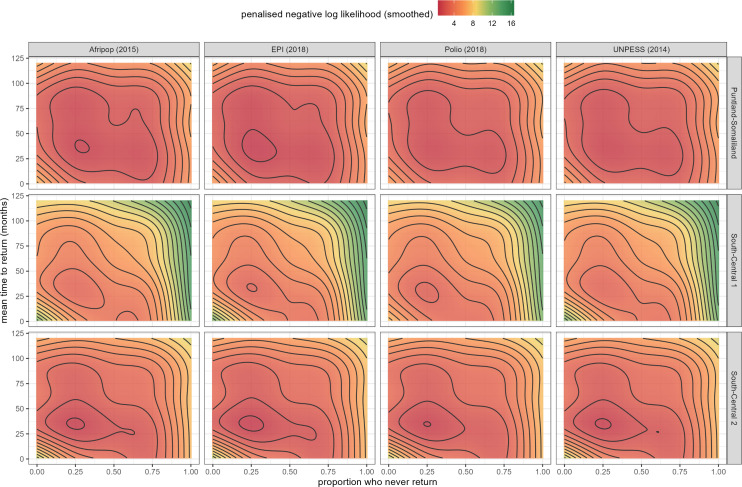
Two-dimensional penalised log-likelihood surfaces for the two unknown parameters, by population source and geographical stratum. Regions coloured dark red denote likely values, and vice versa. Contour lines indicate sets of parameter values with the same likelihood.

Maximum-likelihood estimates of the proportion of IDPs who never return from displacement and the time to return among those who do were similar across strata and population sources, at about one in four and nearly three years, respectively (Table 1). The likelihood surface for Puntland-Somaliland was however relatively uninformative, with large regions of nearly equivalent goodness-of-fit (Fig 4).

**Table 1:** Best estimates of the proportion of IDPs who never return, and the mean time to return among those who do, by base population source and geographical stratum.

Population source	Stratum	Proportion who never return (s)	Mean time to return (1/r, months)
Afripop (2015)	Puntland-Somaliland	0.28	36
	South-Central 1	0.24	33
	South-Central 2	0.23	36
EPI (2018)	Puntland-Somaliland	0.28	35
	South-Central 1	0.24	34
	South-Central 2	0.23	36
Polio (2018)	Puntland-Somaliland	0.28	35
	South-Central 1	0.24	30
	South-Central 2	0.23	35
UNPESS (2014)	Puntland-Somaliland	0.28	35
	South-Central 1	0.25	33
	South-Central 2	0.23	35

### Reconstructed population

**Fig 5:**
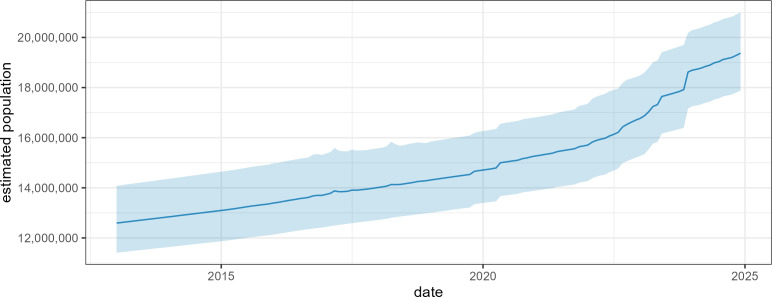
Estimated evolution of the population of Somalia, 2013-2024. The dark line indicates the mean estimate and the shaded area the 95% percentile of simulation outputs.

**Fig 6:**
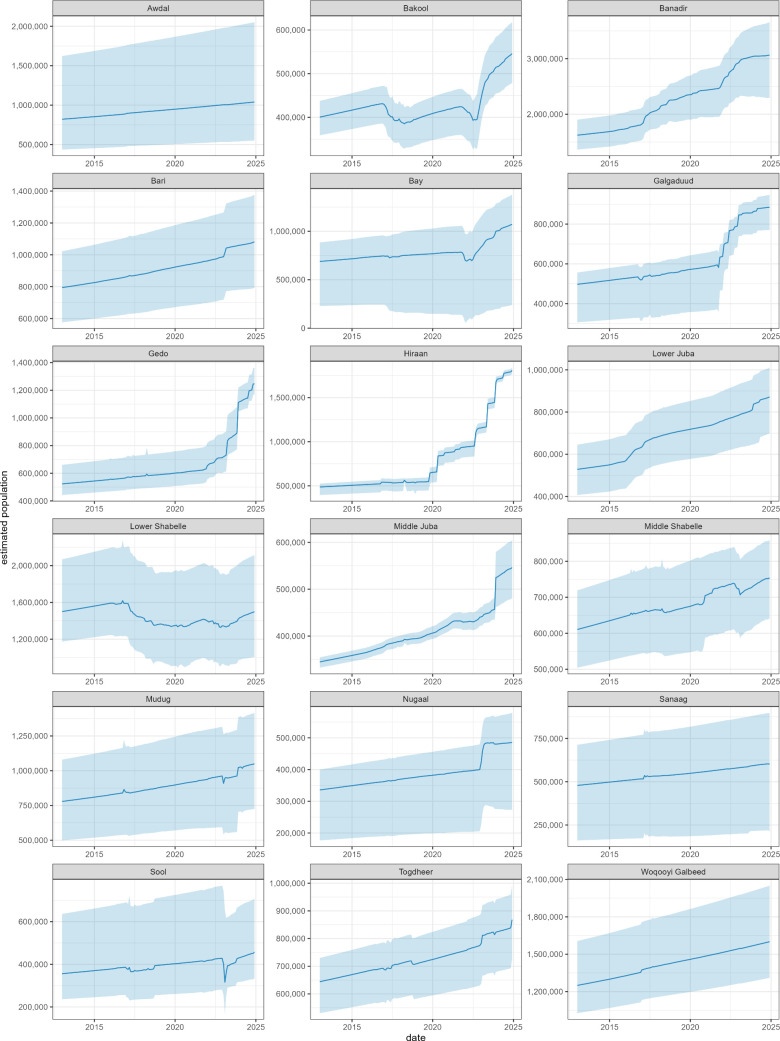
Estimated evolution of the population of Somalia, 2013-2024, by region. The dark line indicates the mean estimate and the shaded area the 95% percentile of simulation outputs.

The model estimated that Somalia’s population rose from about 11.3M in January 2013 to 19.4M by December 2024, though the 95% percentile interval of predictions spanned about ±2M (Fig 5). Variability in the base population estimates meant that, at the regional level, relative uncertainty was even greater (Fig 6). Most but not all regional populations increased, with Banadir’s approximately doubling and nearby regions that IDPs have largely left from, including Bay, Bakool, Lower Shabelle and Middle Shabelle, seeing minimal or negative growth.

An additive model of SMART surveys suggested that period was significantly associated with the proportion of people aged under 5yrs, but the predicted $\pi_{\text{u}\text{5},i,t}$ remained around 24–26% over time, about 5% higher than the SHDS estimate. Model performance was evaluated using leave-one-out cross-validation (LOOCV) [23], yielding a root mean square error (RMSE) [24] of 0.026 ± 0.006. Pearson’s correlation coefficient for the period effect was 0.76, indicating a strong association. Accordingly, Somalia was estimated to have either 5.0M or 4.0M children aged under 5yrs by the end of the period, depending on the proportion used (SMART versus SHDS, respectively).

**Fig 7:**
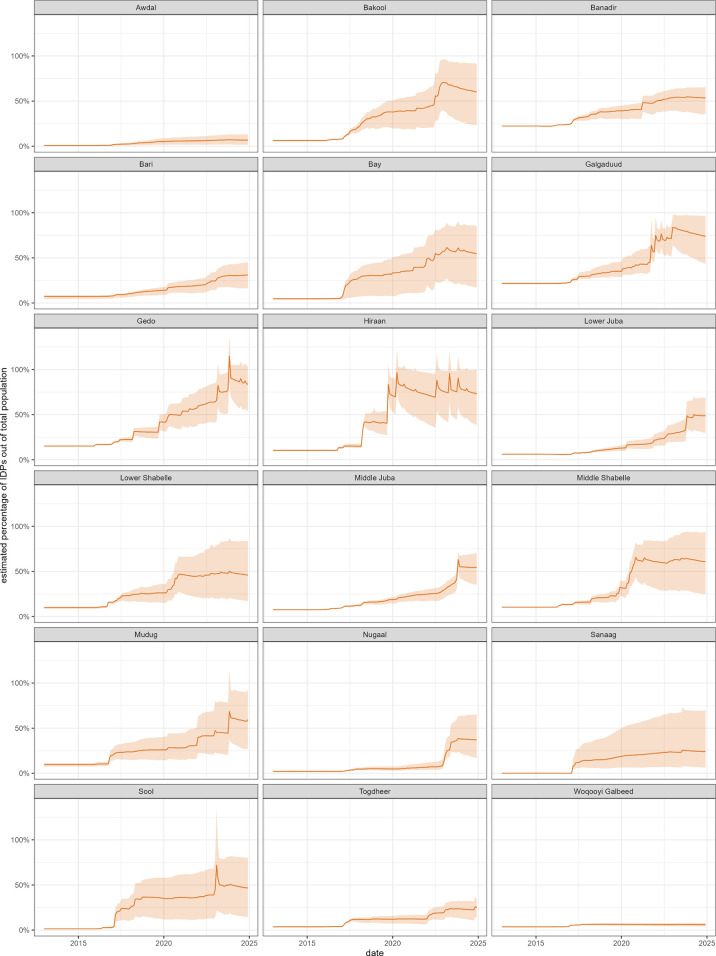
Estimated evolution of the proportion of IDPs among the entire population, 2013-2024, by region. The dark line indicates the mean estimate and the shaded area the 95% percentile of simulation outputs.

Despite the model featuring returns, the number of IDPs was still projected to increase in absolute terms (from 996,000 in January 2013–9,264,000 in December 2024, or 8% to 47% as a proportion of the population). IDP proportions varied considerably by region, with a majority of the population predicted to be displaced in Bakool, Banadir, Bay, Galgaduud, Gedo, Hiraan and Middle Shabelle (Fig 7).

## Discussion

We aimed to reconstruct population change in Somalia at a sub-regional level while propagating the known uncertainty about the population itself and the extent to which and when displaced people left and returned to their communities of origin. To our knowledge, these are the first set of demographic estimates for Somalia that explicitly take into account displacement dynamics. The analysis integrates a variety of datasets into a single modelling framework and suggests a generalisable approach to estimating population in contexts with high levels of displacement and, as is often the case, insufficient data on IDP returns. This work contributes to ongoing efforts to take advantage of statistical and mechanistic models to better measure and predict displacement [25,26]. A comparison between the previous simple method [5], which does not take into account IDP and returnees’ movements, and this method can be found in the S1 Appendix.

Little evidence was found with which to compare our estimates of unknown return parameter estimates, a critical input for the model. In a random sample of displaced people in Mogadishu, most had been displaced for more than 3 years [27]. A national survey found an average duration of displacement of 3 years in 2019, close to our estimates for IDPs who do eventually return home [28]. Most of the evidence on the s parameter consists of stated intentions to stay or return: these appear to vary considerably across Somalia [29], with permanent displacement perhaps more likely among urban IDPs [28]. In south-central Somalia, 68% of nomadic IDPs intended to return [30], a finding corroborated by an earlier study of IDPs in Mogadishu (62%), but in Hargeisa, Somaliland, only 2% of IDPs in the area wished to return, compared with 33% of IDPs in south-central Somalia [31]. In a 2019 national sample, only 23% said they intended to return, with no difference according to the reason for displacement [32].

Exploratory analysis of the predictors associated with return does not allow for any definitive conclusions to be drawn (conf. S1 Appendix for more details). Of the predictors analysed, only terms of trade (goat to cereal) appeared to have the plausibly expected positive association with favourable conditions for IDP return. There was reasonable consistency between different models applied in the analysis. In a reanalysis of countrywide survey data, only the level of humanitarian assistance was weakly associated with stay or return intentions [32].

## Limitations

Our model makes important simplifications of IDP dynamics that largely reflect the scarcity of available data. It is likely that the return parameters we estimate, as well as other inputs such as the natural growth rate, differ substantially at a more granular level than the three strata we pre-specified. The model does not capture the nuance of households separating, for example with some members (e.g., adult men) returning to areas of origin or moving abroad in an attempt to diversify livelihood sources [33]. The assumption of a time-independent rate of return may also be inappropriate: at least one report suggests that the more protracted urban displacement becomes, the less likely IDPs are to return [16]. Our analysis overlooks the phenomenon of multiple displacements: in 2019 four in five IDPs across Somalia reported being displaced only once [32], which suggests that this is a moderately important limitation for our analysis. The model likewise does not capture migration not due to forced displacement. A countrywide labour survey in 2019 suggested that 61% of internal migrants moved because of natural disaster or insecurity [34], suggesting a proportion may have done so more voluntarily to seek educational or livelihood opportunities.

In addition to the model structure, the accuracy of PRMN and DTM’s data affects the robustness of our estimates. It is possible that IDP and returnee estimates were subject to upward bias: a large government survey of IDP settlements in South-Central Somalia found systematically lower IDP numbers than reported by the Cluster Camp Coordination Mechanism [30]. While the PRMN also collects returnee movement data, it is unclear to what extent these are captured [35]: in practice, the return parameters we estimated may be on top of some returnee movement that is already captured in PRMN data.

The exploratory analysis of return predictors also has several limitations. The associations we investigated may be biased by hidden confounding, warranting the use of a more rigorous causal inference approach and adjustment for potential confounders. The data themselves are likely to feature considerable error due to the method used by the DTM for enumerating IDPs and returnees (key informant report rather than actual ground estimation), and it is possible in particular that the ability of informants to correctly partition people by which years they returned in may have been limited. This misclassification error, plausibly non-systematic, would have biased model coefficients towards the null, i.e., impeded observation of the true associations. Critically, the two periods of return were very long (up to four years): it is likely that both the predictors and the rate of IDP return would have fluctuated considerably within each period, but not necessarily with the same pattern: for example, returns might have been concentrated in parts of the period when the predictors were, at least relatively, at their most theoretically favourable values for return. In other words, our use of average rates of return and predictor values may have obscured associations that would have been visible if the data had been broken down by finer time intervals.

## Conclusion

Our study uses a new mechanistic maximum likelihood estimation framework to better understand population change in a context of mass displacement. Although our approach is promising, it needs to be refined and applied to different contexts. In addition, it is essential to improve the collection of field data on returnee movements in order to improve population models. Despite limitations in data availability and potential biases, our work may help to advance the measurement and prediction of displacement dynamics, while also supporting better service planning and evaluation by government and humanitarian actors. Continued research efforts are essential to address the complex challenges of displacement and inform effective interventions in Somalia and similar contexts.

## Supporting information

S1 Appendix Additional methods, figures and data tables. (DOCX)
