# Supplementary material for: Reconstructing Somalia’s population: A district level analysis
Source: PLOS Glob Public Health. 2025 Sep 25;5(9):e0005215. doi: 10.1371/journal.pgph.0005215 (PMC12463287; doi:10.1371/journal.pgph.0005215)
Supplement: S1 Appendix — (DOCX) [file pgph.0005215.s001.docx]

Appendix

Contents

[Quality Score 2](#_Toc208560137)

[Corollary analysis: variability in the rate of IDP return 3](#_Toc208560138)

[Methodology 3](#_Toc208560139)

[Results: Predictors associated with the rate of IDP return 3](#_Toc208560140)

[Discussion 6](#_Toc208560141)

[Influence of growth rate on the population estimation 6](#_Toc208560142)

[Comparison between methodologies 7](#_Toc208560143)

## Quality Score

Each population source has been attributed to a quality score based on the published criteria available on the following website: https://researchonline.lshtm.ac.uk/id/eprint/4668594/.

| **Source** | **UNPESS** | | **Polio** | | **EPI** | | **AfriPop** | |
| --- | --- | --- | --- | --- | --- | --- | --- | --- |
| **Population (millions)** | 12.3 | | 15.8 | | 14.9 | | 11.7 | |
| **criterion** | **score** | **weight** | **score** | **weight** | **score** | **weight** | **score** | **weight** |
| Choice of method | 0.80 | 0.25 | 0.80 | 0.25 | 0.40 | 0.25 | 0.60 | 0.25 |
| Precision* | 0.80 | 0.10 | 0.00 | 0.10 | 0.00 | 0.10 | 0.80 | 0.10 |
| Bias | 0.80 | 0.15 | 0.40 | 0.15 | 0.40 | 0.15 | 0.80 | 0.15 |
| Expertise/credibility | 1.00 | 0.10 | 1.00 | 0.10 | 1.00 | 0.10 | 1.00 | 0.10 |
| Timing of data collection | 0.40 | 0.15 | 0.80 | 0.15 | 0.80 | 0.15 | 0.40 | 0.15 |
| Population included in estimation | 1.00 | 0.15 | 0.40 | 0.15 | 0.40 | 0.15 | 0.40 | 0.15 |
| Plausibility | 0.60 | 0.10 | 0.40 | 0.10 | 0.60 | 0.10 | 0.80 | 0.10 |
| **Total score** | **0.77** | | **0.58** | | **0.50** | | **0.65** | |
|  |  |  |  |  |  |  |  |  |
| *none of the estimates have a confidence interval |  |  |  |  |  |  |  |  |

## Corollary analysis: variability in the rate of IDP return

### Methodology

It is plausible that $r$ (and perhaps $s$) vary as a function of the suitability of settlement conditions in the district of refuge or how favourable conditions for return are in the district of origin. Thus, the average parameter value over the country and period may be expressed as the mean of district- and time-specific values that are themselves a function of place- and time-varying predictors $x_{1},x_{2}\ldots$ :

$$\bar{r}=\frac{1}{I}\sum_{i=1}^{i=K} \sum_{j=1}^{j=K} \sum_{t=1}^{t=T} I_{ij,t}r_{ji,t}\text{ , where } r_{ji,t}\sim\left\{ x_{1,i,t},x_{2,i,t}\ldots\right\}$$

Specifically, there is very strong evidence that IDPs in Somalia leave their homes overwhelmingly due to armed conflict and/or drought (18–20). Of some 22,000 IDP households sampled in 2022, 23% had moved due to conflict and 70% drought (21). Security was a top concern for return (22). We hypothesised that improvements in these factors would encourage people to return to their districts of origin. Thus, we computed the district-specific mean value of key variables for these factors (the number of insecurity events, number of insecurity fatalities, terms of trade when purchasing staple cereal with an average daily wage, terms of trade when bartering a local-quality goat for staple cereal, and rainfall deviation from the historical mean), as well as the mean number of returning IDPs per month over the periods for which DTM data provided district-level stratification of returnee numbers. We then explored the association between each predictor and the rate of IDP return by period through the following alternative model: (i) a generalised linear mixed model with the number of returnees as the outcome, log period duration as offset, district as a random effect, categorical versions of the predictors, and a quasi-Poisson distributional assumption; (ii) a generalised additive mixed model as above but with polynomial B-spline terms for each predictor treated continuously; (iii) a Bayesian kernel machine regression (BKMR) model with the return rate as a continuous variable, assumed to be normally distributed, and 10,000 iterations. BKMR copes well with multicollinearity and is helpful to visualize the dose-response associations between each predictor and the outcome (23). While results are shown below, predictor-dependent variability in return parameters was ultimately not incorporated into the analysis as predictive models were insufficiently accurate (data not shown).

### Results: Predictors associated with the rate of IDP return

Available DTM data included 1,826,197 IDPs and 780,747 returnees across 50 and 74 districts respectively. For 705,416 returnees there was information on how many had returned during at least two specific periods in the past, though in most instances the two periods spanned several years (see Panel A*,* Fig A). As shown in Panel C, Fig A the rate of return was higher during the more recent period 2 than period 1 across the entire dataset, but there was no consistent trend within each district.

The exploratory models we applied yielded few significant associations, and some of these appeared counterintuitive. A generalised linear mixed model suggested that increasing insecurity and improving terms of trade (goat to staple cereal but not wage to staple cereal) were associated with higher returns, while the association with rainfall appeared non-linear (Table A).

Table A. Fixed effect coefficients and p-values of a generalised linear mixed model of returnees per month. All predictor values refer to the district of potential return.

| Term | RR† | 95%CI | p-value |
| --- | --- | --- | --- |
| Insecurity events per month | | | |
| < 1.00 | 1.00 | [reference cat.] |  |
| 1.00 to 1.99 | 1.27 | 0.64 to 2.49 | 0.492 |
| ≥ 2.00 | 2.17 | 1.19 to 3.95 | 0.011* |
| Terms of trade (wage) | | | |
| < 20,000 | 1.00 | [reference cat.] |  |
| 20,000 to 29,999 | 0.86 | 0.47 to 1.59 | 0.642 |
| ≥ 30,000 | 0.92 | 0.48 to 1.76 | 0.810 |
| Terms of trade (goat) | | | |
| < 200,000 | 1.00 | [reference cat.] |  |
| 200,000 to 299,999 | 1.35 | 0.73 to 2.52 | 0.338 |
| ≥ 300,000 | 2.65 | 1.35 to 5.22 | 0.005** |
| Standardised precipitation index | | | |
| below -0.25 SD | 1.00 | [reference cat.] |  |
| -0.25 to -0.01 SD | 0.53 | 0.25 to 1.11 | 0.094 |
| 0.00 to 0.24 SD | 0.43 | 0.22 to 0.85 | 0.015* |
| at or above 0.25 SD | 1.17 | 0.52 to 2.61 | 0.703 |

† Rate ratio

Broadly similar associations were suggested by a generalised additive mixed model, with weak evidence for a negative association between return and terms of trade (wage) (Table B).

Table B. Fixed effect smoothed coefficients and p-values of a generalised additive mixed model of returnees per month. All predictor values refer to the district of potential return.

| Term | RR † | 95%CI | p-value |
| --- | --- | --- | --- |
| Insecurity events per month | 1.32 | 0.82 to 2.11 | 0.252 |
| Terms of trade (goat) | 1.72 | 1.02 to 2.90 | 0.045* |
| Terms of trade (wage) | 0.65 | 0.42 to 1.01 | 0.059 |
| Standardised precipitation index | 1.58 | 0.34 to 7.36 | 0.560 |

† Rate ratio


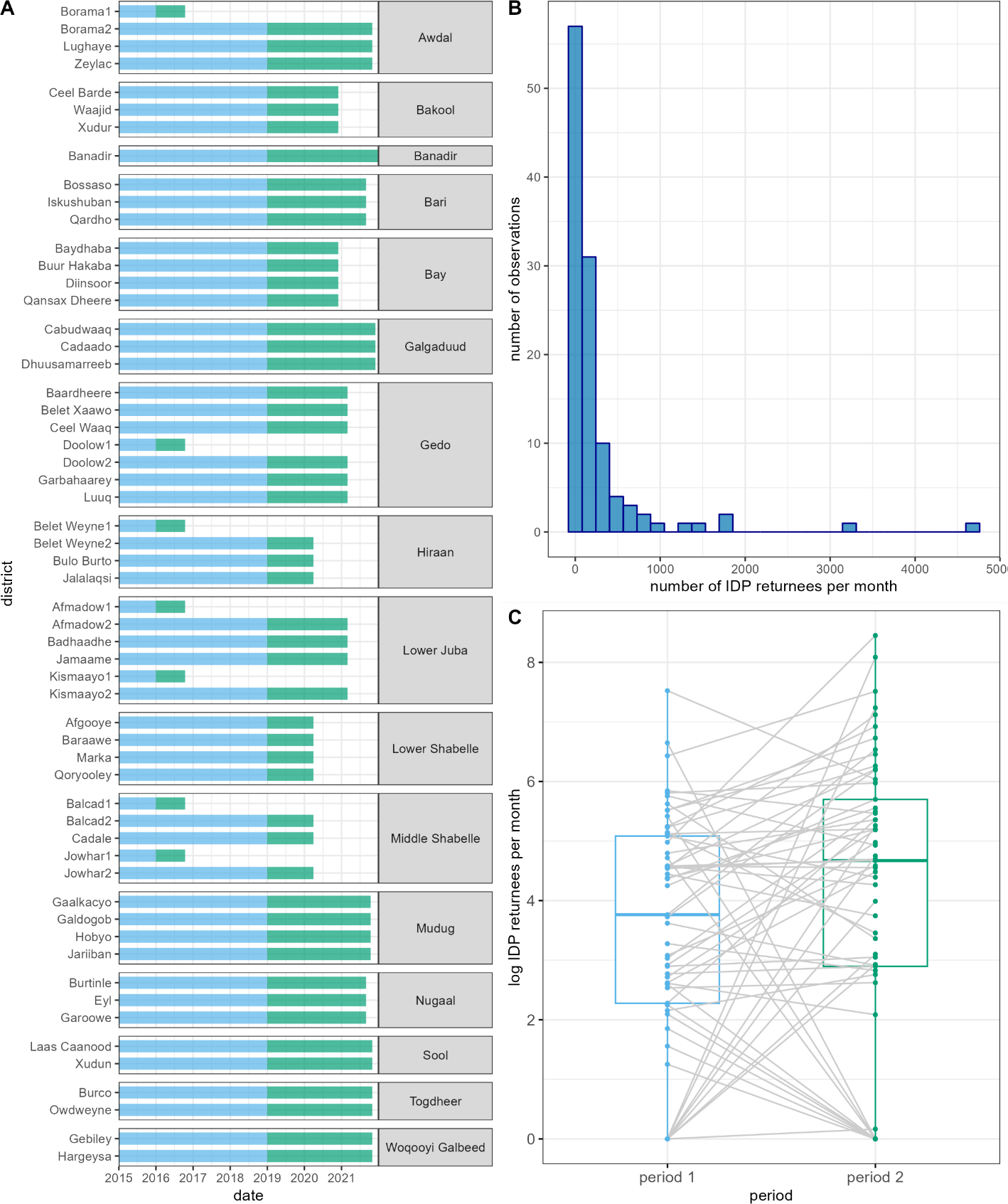


Fig A. Characteristics of the 57 DTM instances analysed. Panel A shows, by region and district, the timespan of the two periods of return available in the dataset. Panel B shows the distribution of the rate of IDP returns per month, irrespective of period. Panel C shows the rate of return per month (logged for readability) during period 1 and period 2, with boxplots showing the median and inter-quartile period, and grey lines connecting the observations for period 1 to period 2 for a given district- assessment instance.

BKMR regression also suggested unclear relationships of insecurity with the outcome, with only terms of trade (goat) showing a clear positive linear association (Fig B). Posterior inclusion probabilities, a measure of variable importance, were 81% for terms of trade (goat), 69% and 67% for insecurity events and fatalities respectively, 55% for rainfall and 50% for terms of trade (wage).


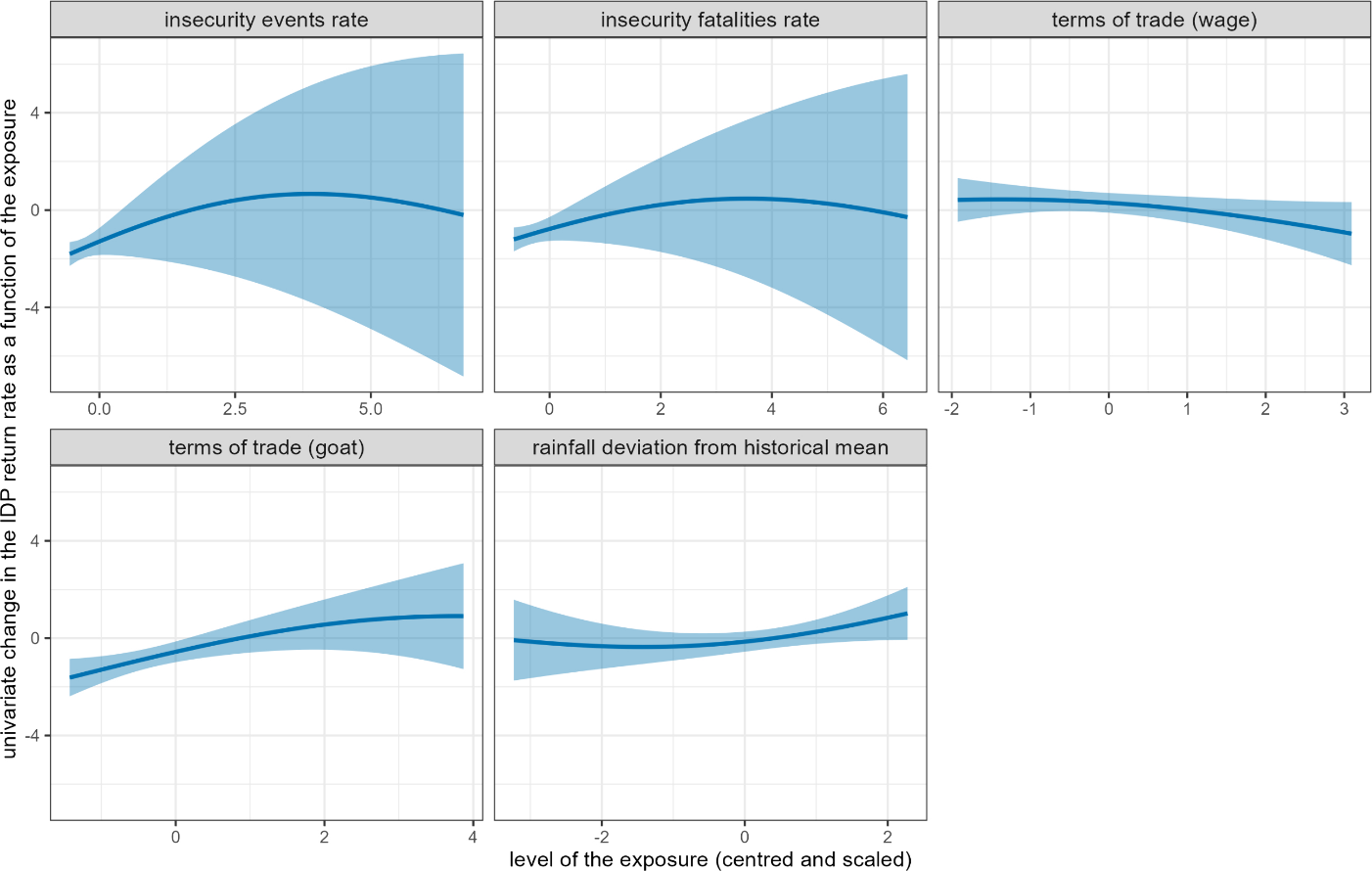


Fig B. Univariate dose-response relationships between each predictor (scaled and centred) and the outcome (IDP return rate), as estimated by Bayesian kernel machine regression.

### Discussion

The exploratory analysis of predictors associated with return does not allow for definitive conclusions. While drought is identified as a major driver of displacement, the influence of seasonality on IDP returns was not explicitly addressed. Future work could incorporate predictors more directly related to agricultural cycles and drought impacts, such as NDVI or other vegetation indices. Additionally, exploring causal relationships would be valuable, as insecurity may both influence and be influenced by drought-related displacement and food insecurity.

## Influence of growth rate on the population estimation

To understand the impact of growth rate assumptions on the population estimates, we applied the reconstruction model described in the methodology using varying growth rate scenarios. Specifically, we explored the effect of decreasing and increasing the baseline growth rate (g), as defined by the SHDS 2020, by 50%. The table below summarizes the resulting population estimates for Somalia as of December 2024.

Table C. Influence of the growth rate parameter on the estimated population in Somalia (December 2024).

| Growth rate scenario | Estimated population (95%CI) |
| --- | --- |
| Baseline growth rate (g) | 19,300,000 (CI: 13,900,000 – 26,300,000) |
| 50% decrease in growth rate (0.5g) | 16,900,000 (CI: 12,300,000-23,300,000) |
| 50% increase in growth rate (1.5g) | 23,100,000 (CI: 18,300,000-28,200,000) |

These results demonstrate that even with substantial variation in the assumed growth rate (±50%), the estimated populations remain within the baseline confidence interval. This suggests that the population reconstruction is robust to plausible changes in growth assumptions, lending confidence to the overall estimate for Somalia in December 2024.

## Comparison between methodologies

Comparison of mean population estimates from two approaches: (i) the previous model, which applied a simple methodology that did not account for internally displaced persons (IDPs) or returnees, and (ii) the new model, which incorporates displacement dynamics as described in this paper. The previous model shows noticeable population declines in some regions (e.g., Lower Shabelle, Bay), largely because returnee movements were not accounted for.


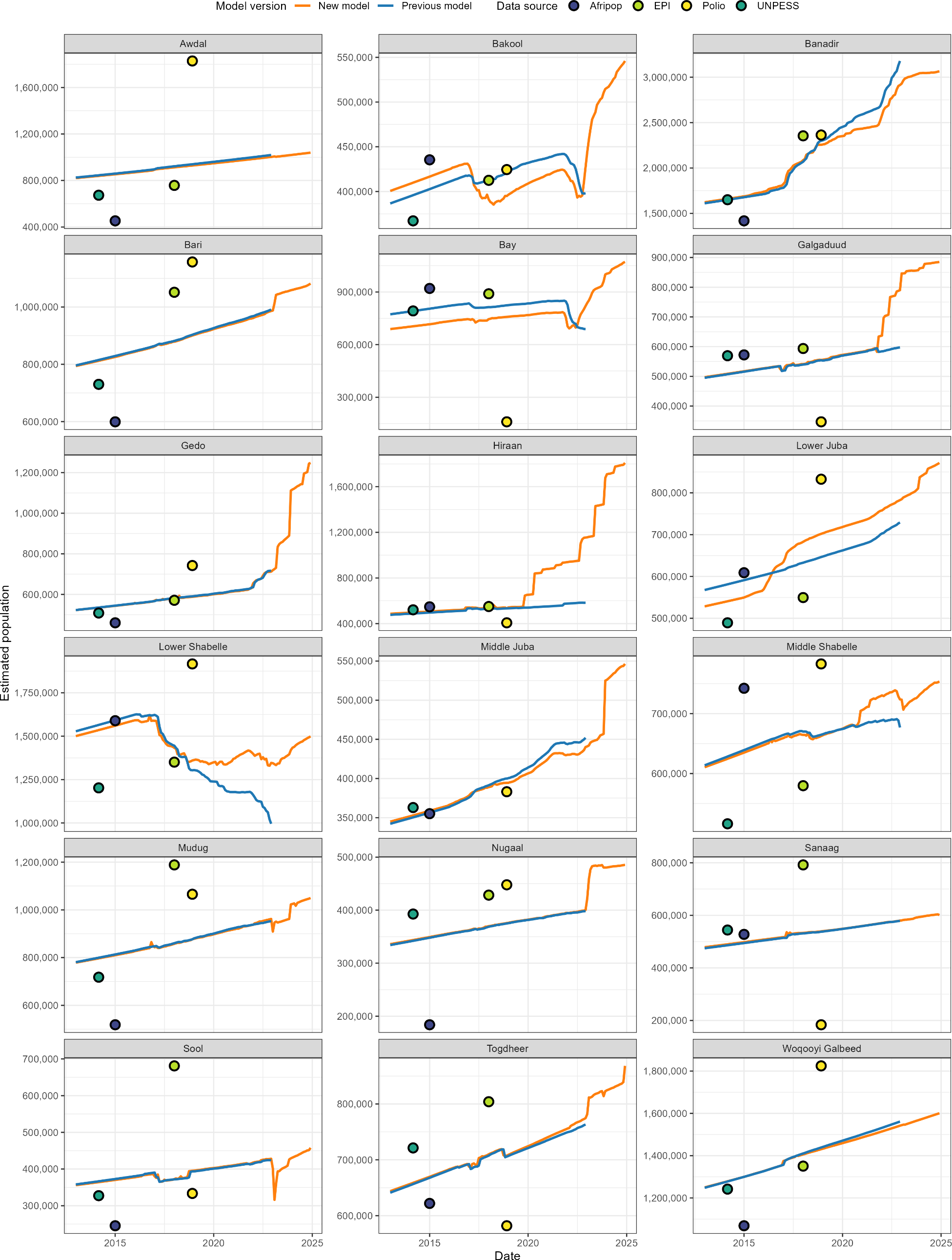


Fig C. Comparison between previous and new population reconstruction models.
